# Supplementary material for: Association between coronary artery disease and incident cancer risk: a systematic review and meta-analysis of cohort studies
Source: PeerJ. 2023 Feb 23;11:e14922. doi: 10.7717/peerj.14922 (PMC9968460; doi:10.7717/peerj.14922)
Supplement: Supplemental Information 3 [file peerj-11-14922-s003.docx]

1. **The rationale for conducting the systematic review**

Cancers and coronary artery disease (CAD) are closely linked with shared risk factors. Some studies have demonstrated increased incidence of CAD after cancer diagnosis. In contrast, several recent clinical and epidemiological studies have found a link between myocardial infarction and new-onset cancer; however, the findings were inconsistent and contradictory. Some of the previous review’s analytic findings were based on only two or three studies and only included patients with myocardial infarction, not all CAD patients. Thus, coronary artery disease’s potential as a causal factor in cancer is still unknown. So we conducted a comprehensive systematic review to update the relationship between CAD and incident cancer.

The rationale for conducting the current systematic review and meta-analysis can be found on pages 4-5 (see the introduction).

1. **The contribution that the systematic review makes to knowledge in light of previously published related reports, including other meta-analyses and systematic reviews**

Based on our analysis of newly published data, we observed an increased risk of incident cancer after a CAD event. This was seen in both males and females, myocardial infarction and non-myocardial infarction subjects, particularly for lung and colorectal cancer. Although this trend may be attributable to several common risk factors and underlying pathophysiologic mechanisms, patients with CAD are still more likely to develop cancer. Close cancer surveillance and possible interventions in the CAD population should be implemented to reduce cancer-related morbidity and mortality.

Please see the last paragraph of the introduction (page 5), and the discussion and conclusion sections for information about the contribution of the current systematic review.
