# Supplementary material for: Association between coronary artery disease and incident cancer risk: a systematic review and meta-analysis of cohort studies
Source: PeerJ. 2023 Feb 23;11:e14922. doi: 10.7717/peerj.14922 (PMC9968460; doi:10.7717/peerj.14922)
Supplement: Supplemental Information 4 [file peerj-11-14922-s004.pdf]

Supplementary File

Association between coronary artery disease and incident cancer risk: a systematic review and meta-analysis of cohort studies

Supplementary Tables and Figures

Supplemental Table S1. Search strategy
Supplemental Tables S2. Newcastle-Ottawa scale for assessment of quality of included studies – Cohort studies
Supplemental Figure S1. Forest plot of incident cancer risk, comparing participants with CAD as those without CAD, with subgroup analysis by country.
Supplemental Figure S2. Forest plot of incident cancer risk, comparing participants with CAD as those without CAD, with subgroup analysis by CAD type.
Supplemental Figure S3. Forest plot of incident cancer risk, comparing participants with CAD as those without CAD by individual cancer type
Supplemental Figure S4. Forest plot of incident cancer risk with follow-up time more than one year, comparing participants with CAD as those without CAD.
Supplemental Figure S5. The Funnel plot of the standard error by odds ratio of the studies included in the meta-analysis.

Table S1. Search strategy

Search strategy in Pubmed

#1	("Coronary Disease"[MeSH Terms] OR coronary heart[Title] OR Coronary Thrombosis[MeSH Terms] OR (Coronary[Title] AND ("thrombosis"[Title] OR "Thromboses"[Title])) OR coronary artery[Title] OR "CAD"[Title] OR coronary atherosclerotic heart disease[Title] OR "CAHD"[Title] OR "arteriosclerosis"[MeSH Terms] OR "arterioscleros*"[Title] OR "atherosclerosis"[MeSH Terms] OR "Atherogenesis"[Title] OR ("atherosclerosis"[Title] OR "atheroscleroses"[Title]) OR Myocardial Infarction[MeSH Terms] OR "Anterior Wall Myocardial Infarction"[MeSH Terms] OR "Myocardial Ischemia"[MeSH Terms] OR (("Heart"[Title] OR "cardiac"[Title] OR "myocardium"[Title] OR "myocardial"[Title] OR syndrome[ti])
#2	("Neoplasms"[MeSH Terms] OR ("Neoplasms"[Title/Abstract] OR (("neoplasm"[Title/Abstract]) OR "Neoplasia"[Title/Abstract]) OR "Neoplasias"[Title/Abstract] OR "Tumors"[Title/Abstract] OR "Tumor"[Title/Abstract] OR "Cancer"[Title/Abstract] OR "Cancers"[Title/Abstract] OR "Malignancy"[Title/Abstract] OR "Malignancies"[Title/Abstract] OR "malignant neoplasm*"[Title/Abstract] OR "malignant cancer"[Title/Abstract] OR "malignant tumors"[Title/Abstract] OR "Hamartoma"[MeSH Terms] OR "Leukemia"[MeSH Terms] OR "Lymphoma"[MeSH Terms] OR "Carcinoma"[MeSH Terms] OR "Adenocarcinoma"[MeSH Terms] OR "sarcoma"[MeSH Terms] OR "Hamartoma"[Title/Abstract] OR "Leukemia"[Title/Abstract] OR "Lymphoma"[Title/Abstract] OR "Carcinoma"[Title/Abstract] OR "Adenocarcinoma"[Title/Abstract] OR "sarcoma"[Title/Abstract]))
#3	("Cohort Studies"[MeSH Terms] OR ("Cohort"[Title/Abstract] OR "incidence studies"[Title/Abstract] OR "incidence study"[Title/Abstract]) OR "Cohort Effect"[MeSH Terms] OR ("cohort effect*"[Title/Abstract] OR "generation effect*"[Title/Abstract]) OR "Follow-Up Studies"[MeSH Terms] OR ("follow up"[Title/Abstract] OR "Followup"[Title/Abstract] OR "follow up"[Title/Abstract]) OR "Longitudinal Studies"[MeSH Terms] OR "Longitudinal"[Title/Abstract] OR "Prospective Studies"[MeSH Terms] OR "prospective"[Title/Abstract] OR "Retrospective Studies"[MeSH Terms] OR "retrospect*"[Title/Abstract] OR "Observational Study"[Publication Type] OR "Observational Studies as Topic"[MeSH Terms] OR "Epidemiologic Studies"[MeSH Terms] OR "Case-Control Studies"[MeSH Terms] OR ("Case-Control"[Title/Abstract] OR "Case-Comparison"[Title/Abstract] OR "Case-Compeer"[Title/Abstract] OR "Case-Base"[Title/Abstract]))
#4	#1 AND #2 AND #3

search detail	("Coronary Disease"[MeSH Terms] OR coronary heart[Title] OR Coronary Thrombosis[MeSH Terms] OR (Coronary[Title] AND ("thrombosis"[Title] OR "Thromboses"[Title])) OR coronary artery[Title] OR "CAD"[Title] OR coronary atherosclerotic heart disease[Title] OR "CAHD"[Title] OR "arteriosclerosis"[MeSH Terms] OR "arterioscleros*"[Title] OR "atherosclerosis"[MeSH Terms] OR "Atherogenesis"[Title] OR ("atherosclerosis"[Title] OR "atheroscleroses"[Title]) OR Myocardial Infarction[MeSH Terms] OR "Anterior Wall Myocardial Infarction"[MeSH Terms] OR "Myocardial Ischemia"[MeSH Terms] OR (("Heart"[Title] OR "cardiac"[Title] OR "myocardium"[Title] OR "myocardial"[Title] OR syndrome[ti]) AND ("infarct"[Title] OR "infarction"[Title] OR "attack"[Title] OR "ischemia"[Title] OR "ischemic"[Title] OR ischaemic[title] OR "atherosclero*"[Title])) OR ("Coronary"[Title] AND ("thrombosis"[Title] OR "Thromboses"[Title] OR "atherosclero*"[Title]))) AND ("Neoplasms"[MeSH Terms] OR ("Neoplasms"[Title/Abstract] OR (("neoplasm"[Title/Abstract]) OR "Neoplasia"[Title/Abstract]) OR "Neoplasias"[Title/Abstract] OR "Tumors"[Title/Abstract] OR "Tumor"[Title/Abstract] OR "Cancer"[Title/Abstract] OR "Cancers"[Title/Abstract] OR "Malignancy"[Title/Abstract] OR "Malignancies"[Title/Abstract] OR "malignant neoplasm*"[Title/Abstract] OR "malignant cancer"[Title/Abstract] OR "malignant tumors"[Title/Abstract] OR "Hamartoma"[MeSH Terms] OR "Leukemia"[MeSH Terms] OR "Lymphoma"[MeSH Terms] OR "Carcinoma"[MeSH Terms] OR "Adenocarcinoma"[MeSH Terms] OR "sarcoma"[MeSH Terms] OR "Hamartoma"[Title/Abstract] OR "Leukemia"[Title/Abstract] OR "Lymphoma"[Title/Abstract] OR "Carcinoma"[Title/Abstract] OR "Adenocarcinoma"[Title/Abstract] OR "sarcoma"[Title/Abstract])) AND ("Cohort Studies"[MeSH Terms] OR ("Cohort"[Title/Abstract] OR "incidence studies"[Title/Abstract] OR "incidence study"[Title/Abstract]) OR "Cohort Effect"[MeSH Terms] OR ("cohort effect*"[Title/Abstract] OR "generation effect*"[Title/Abstract]) OR "Follow-Up Studies"[MeSH Terms] OR ("follow up"[Title/Abstract] OR "Followup"[Title/Abstract] OR "follow up"[Title/Abstract]) OR "Longitudinal Studies"[MeSH Terms] OR "Longitudinal"[Title/Abstract] OR "Prospective Studies"[MeSH Terms] OR "prospective"[Title/Abstract] OR "Retrospective Studies"[MeSH Terms] OR "retrospect*"[Title/Abstract] OR "Observational Study"[Publication Type] OR "Observational Studies as Topic"[MeSH Terms] OR "Epidemiologic Studies"[MeSH Terms] OR "Case-Control Studies"[MeSH Terms] OR ("Case-Control"[Title/Abstract] OR "Case-Comparison"[Title/Abstract] OR "Case-Compeer"[Title/Abstract] OR "Case-Base"[Title/Abstract]))

Search strategy in Embase

No.	Query
#71	#70 AND [embase]/lim NOT ([embase]/lim AND [medline]/lim)

#70	#68 NOT #69
#69	#68 AND ('chapter'/it OR 'conference abstract'/it OR 'editorial'/it OR 'erratum'/it OR 'letter'/it OR 'note'/it OR 'tombstone'/it)
#68	#46 AND #56 AND #67
#67	#57 OR #58 OR #59 OR #60 OR #61 OR #62 OR #63 OR #64 OR #65 OR #66
#66	'incidence'/exp OR 'incidence':ti,ab OR 'incidence rate':ti,ab OR 'rate, incidence':ti,ab
#65	'cohort effect'/exp OR 'cohort effect':ti,ab
#64	'observational study'/exp OR 'observation studies':ti,ab OR 'observation study':ti,ab OR 'observational studies':ti,ab OR 'observational study':ti,ab
#63	'retrospective study'/exp OR 'retrospective design':ti,ab OR 'retrospective study':ti,ab OR 'retrospective studies':ti,ab
#62	'prospective study'/exp OR 'prospective method':ti,ab OR 'prospective studies':ti,ab OR 'prospective study':ti,ab OR 'study, prospective':ti,ab
#61	'longitudinal study'/exp OR 'longitudinal evaluation':ti,ab OR 'longitudinal studies':ti,ab OR 'longitudinal study':ti,ab
#60	'follow up'/exp OR 'follow up':ti,ab OR 'follow up study':ti,ab OR 'follow-up studies':ti,ab OR 'followup':ti,ab OR 'lost to follow up':ti,ab OR 'lost to follow-up':ti,ab
#59	'cohort analysis'/exp OR 'analysis, cohort':ti,ab OR 'cohort analysis':ti,ab OR 'cohort life cycle':ti,ab OR 'cohort studies':ti,ab OR 'cohort study':ti,ab
#58	'case control study'/exp OR 'case control study':ti,ab OR 'case-control studies':ti,ab OR 'case-control study':ti,ab OR 'control study, case':ti,ab OR 'matched case control':ti,ab OR 'matched case control studies':ti,ab OR 'matched case control study':ti,ab
#57	'epidemiology'/exp/mj OR 'epidemiologic studies':ti,ab OR 'epidemiologic research':ti,ab OR 'epidemiology':ti,ab
#56	#47 OR #48 OR #49 OR #50 OR #51 OR #52 OR #53 OR #54 OR #55
#55	'liver cell carcinoma'/exp OR 'hepatocarcinoma':ti OR 'hepatoma':ti

#54	'leukemia'/exp OR 'aleukaemia':ti OR 'aleukemia':ti OR 'leucaemia':ti OR 'leucemia':ti OR 'leukaemia':ti OR 'leukemia':ti
#53	'adenocarcinoma'/exp OR 'adenocancer':ti OR 'adenocarcinoma':ti
#52	'carcinoma'/exp OR 'carcinoma':ti
#51	'sarcoma'/exp OR 'histioblastoma':ti OR 'histiosarcoma':ti OR 'sarcoma':ti
#50	'lymphoma'/exp OR 'adenolymphoma':ti OR 'cystadenoma lymphomatosum':ti OR 'lymphoma':ti OR 'lymphomas':ti
#49	malignancy:ti OR malignancies:ti
#48	'malignant neoplasm'/exp OR 'cancer':ti OR 'cancers':ti OR 'malignant neoplasia':ti
#47	'neoplasm'/exp OR 'neoplasia':ti OR 'neoplasm':ti OR 'neoplasms':ti OR 'neoplastic':ti OR 'tumor':ti OR tumours:ti OR 'tumour':ti
#46	#39 OR #40 OR #41 OR #42 OR #43 OR #44 OR #45
#45	'heart muscle ischemia'/exp OR 'acute heart muscle ischaemia':ti OR 'acute heart muscle ischemia':ti OR 'cardiac ischaemia':ti OR 'cardiac ischemia':ti OR 'cardiac muscle ischaemia':ti OR 'cardiac muscle ischemia':ti OR 'coronary artery ischaemia':ti OR 'coronary artery ischemia':ti OR 'coronary ischaemia':ti OR 'coronary ischemia':ti OR 'coronary syndrome':ti OR 'heart ischaemia':ti OR 'heart ischaemic arrest':ti OR 'heart ischaemic attack':ti OR 'heart ischemia':ti OR 'heart ischemic arrest':ti OR 'heart ischemic attack':ti OR 'heart muscle ischaemia':ti OR 'ischaemic heart':ti OR 'ischaemic heart arrest':ti OR 'ischaemic myocardium':ti OR 'ischemic heart':ti OR 'ischemic myocardium':ti OR 'myocardial ischaemia':ti OR 'myocardial ischemia':ti OR 'myocardium ischaemia':ti OR 'myocardium ischemia':ti
#44	'heart infarction'/exp OR 'cardiac infarct':ti OR 'cardiac infarction':ti OR 'cardial infarct':ti OR 'heart attack':ti OR 'heart infarct':ti OR 'heart infarction':ti OR 'myocardial infarction':ti OR 'myocardial infarct':ti OR 'myocardium infarct':ti OR 'myocardium infarction':ti OR 'acute heart infarction'/exp OR 'acute cardiac infarction':ti OR 'acute heart infarction':ti OR 'acute myocardial infarction':ti OR 'heart infarction, acute':ti

#43	'arteriosclerosis'/exp OR 'arterial sclerosis':ti OR 'arterio-sclerosis':ti OR 'arteriosclerosis':ti OR 'arteriosclerotic disease':ti OR 'arteriosclerotic disorder':ti OR 'arteriosclerotic vascular disease':ti OR 'arteriosclerotic vascular disorder':ti OR 'vascular sclerosis':ti OR 'arteriolosclerosis'/exp OR 'arteriolar sclerosis':ti OR 'arteriolo-sclerosis':ti OR 'arteriolosclerosis':ti OR 'arteriolosclerotic disease':ti OR 'arteriolosclerotic small vessel disease':ti OR 'arteriolosis':ti OR 'atherosclerosis'/exp OR 'atheriosclerotic disease':ti OR 'athero-sclerosis':ti OR 'atheromatous sclerosis':ti OR 'atherosclerosis':ti OR 'atherosclerosis grading':ti OR 'atherosclerotic disease':ti OR 'atherosclerotic disorder':ti OR 'atherosclerotic vascular disease':ti OR 'atherosclerotic vascular disorder':ti
#42	'coronary artery thrombosis'/exp OR 'acute coronary artery thrombosis':ti OR 'arterial thrombosis, coronary':ti OR 'coronary arterial thrombosis':ti OR 'coronary artery thrombosis':ti OR 'coronary thrombosis':ti OR 'thrombosis, coronary artery':ti
#41	'ischemic heart disease'/exp OR 'heart disease, coronary':ti OR 'heart disease, ischaemic':ti OR 'heart disease, ischemic':ti OR 'ischaemia heart disease':ti OR 'ischaemic cardiac disease':ti OR 'ischaemic cardial disease':ti OR 'ischaemic heart disease':ti OR 'ischemia heart disease':ti OR 'ischemic cardiac disease':ti OR 'ischemic cardial disease':ti OR 'ischemic cardiopathy':ti OR 'ischemic heart disease':ti
#40	cad:ti,ab OR cahd:ti,ab
#39	'coronary artery disease'/exp OR 'coronary artery disease':ti OR 'coronary disease':ti OR 'multivessel coronary artery disease':ti
#38	#8 AND #18 AND #37
#37	#27 OR #28 OR #29 OR #30 OR #31 OR #32 OR #33 OR #34 OR #35 OR #36
#36	'incidence'/exp OR 'incidence':ti,ab OR 'incidence rate':ti,ab OR 'rate, incidence':ti,ab
#35	'cohort effect'/exp OR 'cohort effect':ti,ab
#34	'observational study'/exp OR 'observation studies':ti,ab OR 'observation study':ti,ab OR 'observational studies':ti,ab OR 'observational study':ti,ab
#33	'retrospective study'/exp OR 'retrospective design':ti,ab OR 'retrospective study':ti,ab OR 'retrospective studies':ti,ab
#32	'prospective study'/exp OR 'prospective method':ti,ab OR 'prospective studies':ti,ab OR 'prospective study':ti,ab OR 'study, prospective':ti,ab
#31	'longitudinal study'/exp OR 'longitudinal evaluation':ti,ab OR 'longitudinal studies':ti,ab OR 'longitudinal study':ti,ab

#30	'follow up'/exp OR 'follow up':ti,ab OR 'follow up study':ti,ab OR 'follow-up studies':ti,ab OR 'followup':ti,ab OR 'lost to follow up':ti,ab OR 'lost to follow-up':ti,ab
#29	'cohort analysis'/exp OR 'analysis, cohort':ti,ab OR 'cohort analysis':ti,ab OR 'cohort life cycle':ti,ab OR 'cohort studies':ti,ab OR 'cohort study':ti,ab
#28	'case control study'/exp OR 'case control study':ti,ab OR 'case-control studies':ti,ab OR 'case-control study':ti,ab OR 'control study, case':ti,ab OR 'matched case control':ti,ab OR 'matched case control studies':ti,ab OR 'matched case control study':ti,ab
#27	'epidemiology'/exp/mj OR 'epidemiologic studies':ti,ab OR 'epidemiologic research':ti,ab OR 'epidemiology':ti,ab
#26	'observational study'/exp
#25	'retrospective study'/exp
#24	'prospective study'/exp
#23	'longitudinal study'/exp
#22	'evaluation and follow up'/exp
#21	'attack rate'/exp OR 'cancer incidence'/exp
#20	'cohort effect'/exp
#19	'cohort analysis'/exp
#18	#9 OR #10 OR #11 OR #12 OR #13 OR #14 OR #15 OR #16 OR #17
#17	'liver cell carcinoma'/exp OR 'hepatocarcinoma':ti OR 'hepatoma':ti
#16	'leukemia'/exp OR 'aleukaemia':ti OR 'aleukemia':ti OR 'leucaemia':ti OR 'leucemia':ti OR 'leukaemia':ti OR 'leukemia':ti
#15	'adenocarcinoma'/exp OR 'adenocancer':ti OR 'adenocarcinoma':ti

#14	'carcinoma'/exp OR 'carcinoma':ti
#13	'sarcoma'/exp OR 'histioblastoma':ti OR 'histiosarcoma':ti OR 'sarcoma':ti
#12	'lymphoma'/exp OR 'adenolymphoma':ti OR 'cystadenoma lymphomatosum':ti OR 'lymphoma':ti OR 'lymphomas':ti
#11	malignancy:ti OR malignancies:ti
#10	'malignant neoplasm'/exp OR 'cancer':ti OR 'cancers':ti OR 'malignant neoplasia':ti
#9	'neoplasm'/exp OR 'neoplasia':ti OR 'neoplasm':ti OR 'neoplasms':ti OR 'neoplastic':ti OR 'tumor':ti OR tumours:ti OR 'tumour':ti
#8	#1 OR #2 OR #3 OR #4 OR #5 OR #6 OR #7
#7	'heart muscle ischemia'/exp OR 'acute heart muscle ischaemia':ti OR 'acute heart muscle ischemia':ti OR 'cardiac ischaemia':ti OR 'cardiac ischemia':ti OR 'cardiac muscle ischaemia':ti OR 'cardiac muscle ischemia':ti OR 'coronary artery ischaemia':ti OR 'coronary artery ischemia':ti OR 'coronary ischaemia':ti OR 'coronary ischemia':ti OR 'coronary syndrome':ti OR 'heart ischaemia':ti OR 'heart ischaemic arrest':ti OR 'heart ischaemic attack':ti OR 'heart ischemia':ti OR 'heart ischemic arrest':ti OR 'heart ischemic attack':ti OR 'heart muscle ischaemia':ti OR 'ischaemic heart':ti OR 'ischaemic heart arrest':ti OR 'ischaemic myocardium':ti OR 'ischemic heart':ti OR 'ischemic myocardium':ti OR 'myocardial ischaemia':ti OR 'myocardial ischemia':ti OR 'myocardium ischaemia':ti OR 'myocardium ischemia':ti
#6	'heart infarction'/exp OR 'cardiac infarct':ti OR 'cardiac infarction':ti OR 'cardial infarct':ti OR 'heart attack':ti OR 'heart infarct':ti OR 'heart infarction':ti OR 'myocardial infarction':ti OR 'myocardial infarct':ti OR 'myocardium infarct':ti OR 'myocardium infarction':ti OR 'acute heart infarction'/exp OR 'acute cardiac infarction':ti OR 'acute heart infarction':ti OR 'acute myocardial infarction':ti OR 'heart infarction, acute':ti
#5	'arteriosclerosis'/exp OR 'arterial sclerosis':ti OR 'arterio-sclerosis':ti OR 'arteriosclerosis':ti OR 'arteriosclerotic disease':ti OR 'arteriosclerotic disorder':ti OR 'arteriosclerotic vascular disease':ti OR 'arteriosclerotic vascular disorder':ti OR 'vascular sclerosis':ti OR 'arteriolosclerosis'/exp OR 'arteriolar sclerosis':ti OR 'arteriolo-sclerosis':ti OR 'arteriolosclerosis':ti OR 'arteriolosclerotic disease':ti OR 'arteriolosclerotic small vessel disease':ti OR 'arteriolosis':ti OR 'atherosclerosis'/exp OR 'atheriosclerotic disease':ti OR 'athero-sclerosis':ti OR 'atheromatous sclerosis':ti OR 'atherosclerosis':ti OR 'atherosclerosis grading':ti OR 'atherosclerotic disease':ti OR 'atherosclerotic disorder':ti OR 'atherosclerotic vascular disease':ti OR 'atherosclerotic vascular disorder':ti

#4	'coronary artery thrombosis'/exp OR 'acute coronary artery thrombosis':ti OR 'arterial thrombosis, coronary':ti OR 'coronary arterial thrombosis':ti OR 'coronary artery thrombosis':ti OR 'coronary thrombosis':ti OR 'thrombosis, coronary artery':ti
#3	'ischemic heart disease'/exp OR 'heart disease, coronary':ti OR 'heart disease, ischaemic':ti OR 'heart disease, ischemic':ti OR 'ischaemia heart disease':ti OR 'ischaemic cardiac disease':ti OR 'ischaemic cardial disease':ti OR 'ischaemic heart disease':ti OR 'ischemia heart disease':ti OR 'ischemic cardiac disease':ti OR 'ischemic cardial disease':ti OR 'ischemic cardiopathy':ti OR 'ischemic heart disease':ti
#2	cad:ti,ab OR cahd:ti,ab
#1	'coronary artery disease'/exp OR 'coronary artery disease':ti OR 'coronary disease':ti OR 'multivessel coronary artery disease':ti

Search strategy in Cochrane Library

ID	Search
#1	MeSH descriptor: [Coronary Disease] explode all trees+B22B1B7:B21
#2	MeSH descriptor: [Coronary Thrombosis] explode all trees
#3	MeSH descriptor: [Coronary Artery Disease] explode all trees
#4	(coronary AND (thrombosis OR Thromboses OR Arterioscler* OR artery OR heart)):ti
#5	MeSH descriptor: [Arteriosclerosis] explode all trees
v6	MeSH descriptor: [Atherosclerosis] explode all trees
#7	(Arterioscleros* OR Atherogenesis OR atheroscleros* OR atherosclerotic OR CAD OR CAHD):ti
#8	MeSH descriptor: [Myocardial Infarction] explode all trees
#9	MeSH descriptor: [Myocardial Ischemia] explode all trees

#10	MeSH descriptor: [Anterior Wall Myocardial Infarction] explode all trees
#11	((heart OR cardiac OR myocardium OR myocardial OR syndrome) AND (infarct* OR attack OR ischemia OR ischemic OR ischaemi* OR atherosclero*)):ti
#12	#1 OR #2 OR #3 OR #4 OR #5 OR #6 OR #7 OR #8 OR #9 OR #10 OR #11
#13	MeSH descriptor: [Neoplasms] explode all trees
#14	MeSH descriptor: [Sarcoma] explode all trees
#15	MeSH descriptor: [Lymphoma] explode all trees

Search strategy in Airiti library

ID	Search
#1	[ALL3]:(冠心 OR 梗塞 OR 缺血 OR 心肌) AND [ALL3]:(癌 OR 腫瘤)

Tables S2. Newcastle-Ottawa scale for assessment of quality of included studies – Cohort studies (each star represents if individual criterion within the subsection was fulfilled)

Study	Selection				Comparability		Outcome			**Total quality score**
Quality assessment criteria	Representativeness of exposed cohort	Selection of non–exposed cohort	Ascertainment of exposure	Demonstration that outcome of interest was not present at start of study	Adjust for the most important risk factors	Adjust for other risk factors	Assessment of outcome	Follow–up length	Loss to follow–up rate	
Acceptable (★)	Representative of general adult population in community (age/sex/being at risk of disease)	Drawn from the same community as exposed cohort	Secure records, Structured interview	Yes, or excluded when analysis	Yes, at least for age and sex	Yes, and smoking must be included	Independent blind assessment, record linkage	Follow–up >1 years	Follow–up completed, or small subjects lost(<20%), or lost subjects unlikely to introduce bias*	
Rinde, 2017	★	★	★	★	★	★	★	★	★	**9**
Dreyer, 1998	★	★	★	-	-	-	★	★	-	**5**

Malmburg, 2018	★	★	★	★	★	★	★	★	★	**9**
Perhsson, 2005	★	★	★	-	★	-	★	★	-	**6**
Thomas II, 2012	★	★	★	★	★	★	★	★	★	**9**
Suzuki, 2017	★	★	★	★	★	★	★	★	★	**9**
Dzaye, 2021	★	★	★	★	★	★	★	★	★	**9**
Berton , 2018	★	★	★	★	-	-	★	★	-	**6**
Erichsen, 2013	★	★	★	★	★	★	★	★	★	**9**
Kwa, 2020	★	★	★	★	★	★	★	★	★	**9**
Handy, 2016	★	★	★	★	★	★	★	★	★	**9**
Peng, 2020	★	★	★	-	★	★	★	★	-	**7**
Vinter, 2018	★	★	★	★	★	★	★	★	★	**9**
Mirbolouk, 2020	★	★	★	-	★	★	★	★	★	**8**

Figure S1. Forest plot of incident cancer risk, comparing participants with CAD as those without CAD, with subgroup analysis by country. CAD, coronary artery disease; CI, confidence interval; OR, odds ratio; se, standard error; TE, treatment effect.

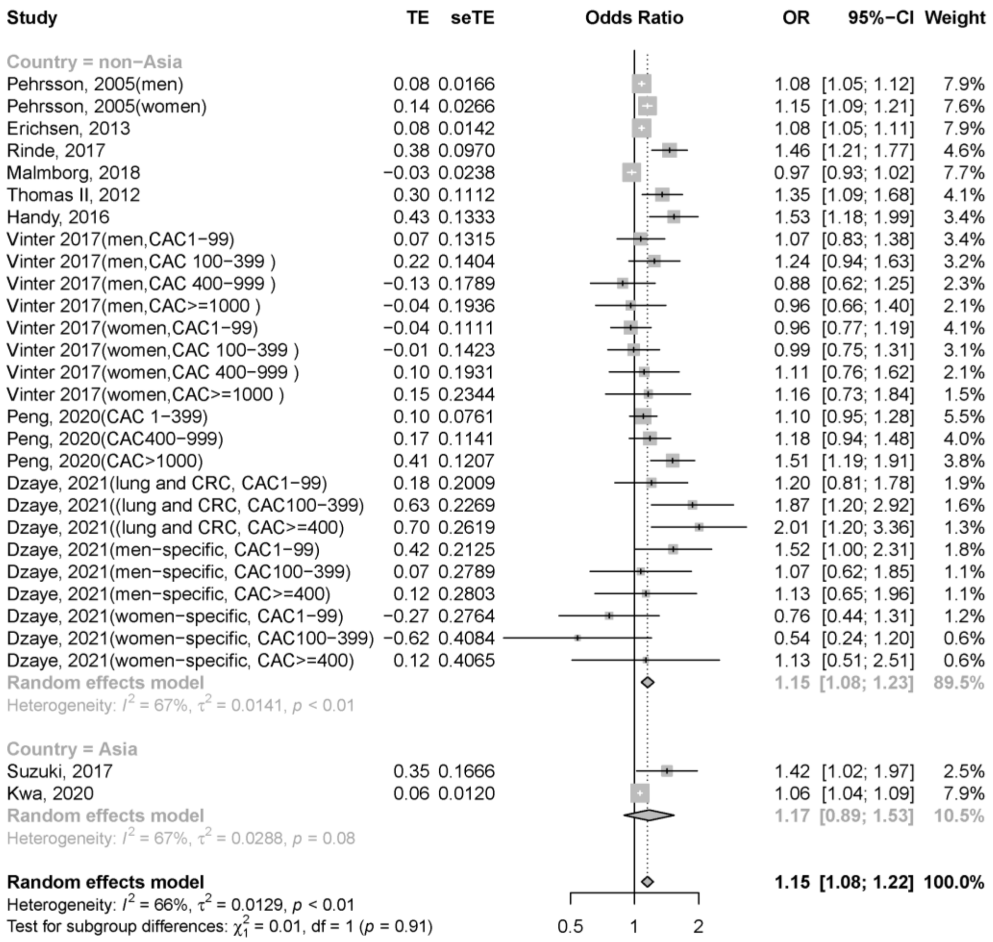

Figure S2. Forest plot of incident cancer risk, comparing participants with CAD as those without CAD, with subgroup analysis by CAD type. CAD, coronary artery disease; CI, confidence interval; MI, myocardial infarction; OR, odds ratio; se, standard error; TE, treatment effect.

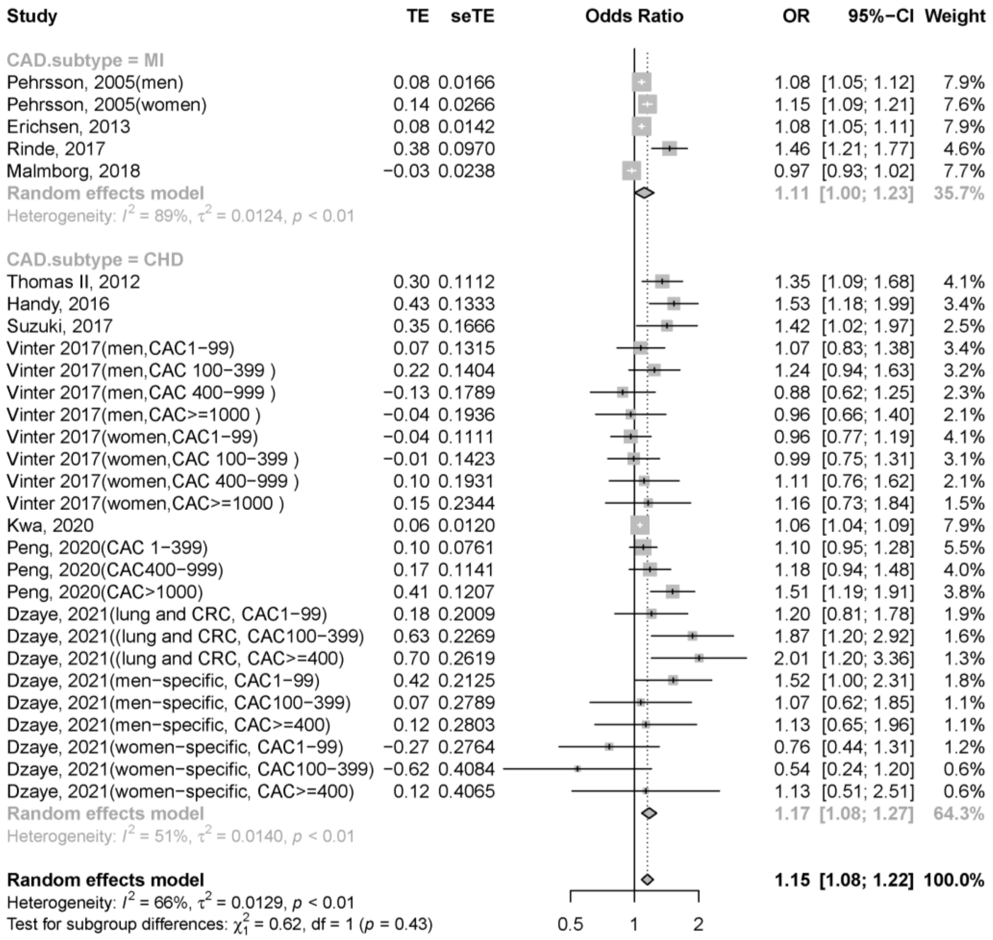

Figure S3. Forest plot of incident cancer risk, comparing participants with CAD as those without CAD by individual cancer type. CAD, coronary artery disease; CI, confidence interval; OR, odds ratio; se, standard error; TE, treatment effect.

(1) Prostate cancer

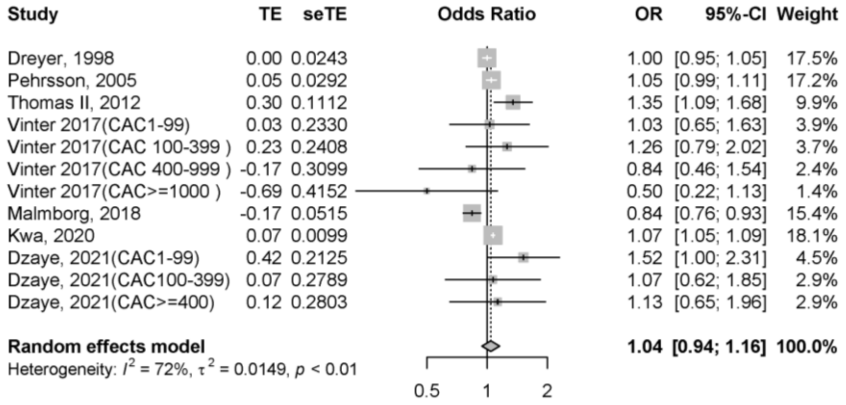

(2) Liver cancer

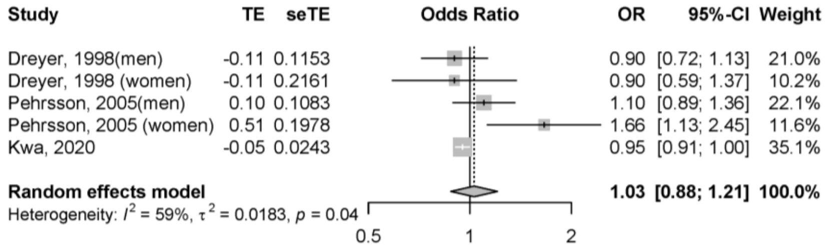

Figure S4. Forest plot of incident cancer risk with follow-up time more than one year, comparing participants with CAD as those without CAD. CAD, coronary artery disease; CI, confidence interval; OR, odds ratio; se, standard error; TE, treatment effect.

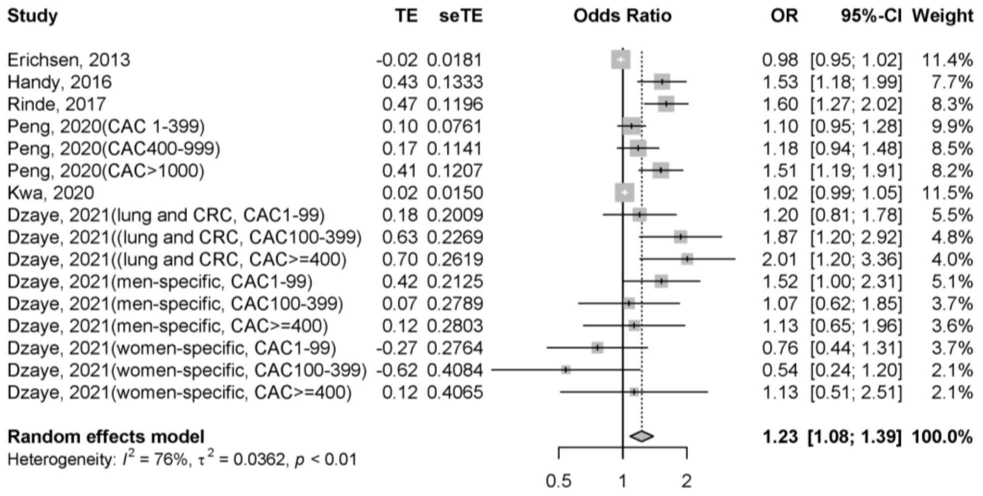

Figure S5. The Funnel plot of the standard error by odds ratio of the studies included in the meta-analysis.

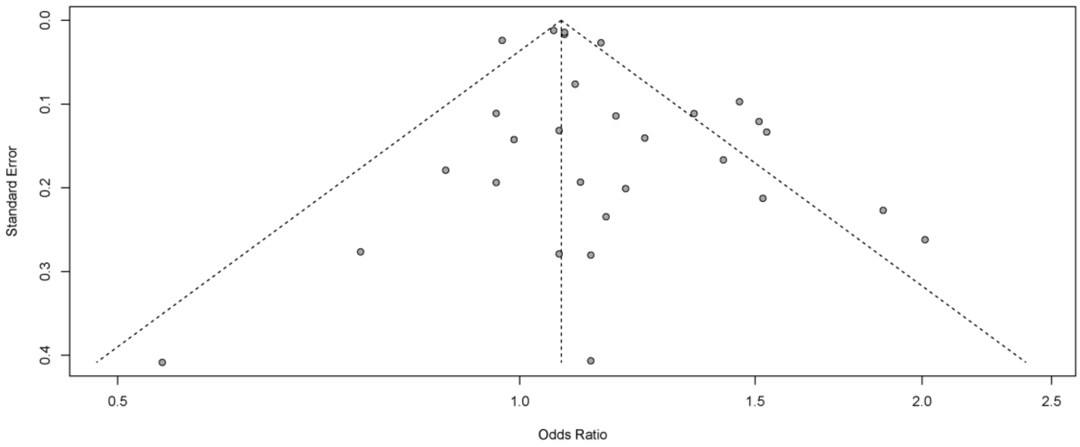

Egger's test, slope = 1.97, p = 0.06
