# Supplementary material for: Association between coronary artery disease and incident cancer risk: a systematic review and meta-analysis of cohort studies
Source: PeerJ. 2023 Feb 23;11:e14922. doi: 10.7717/peerj.14922 (PMC9968460; doi:10.7717/peerj.14922)
Supplement: Supplemental Information 5 [file peerj-11-14922-s005.pdf]

# Association between coronary artery disease and incident cancer risk: a systematic review and meta-analysis of cohort studies

*By* HsinHao Chen


| | | | |
|---|---|---|---|
| WORD COUNT | 5161 | TIME SUBMITTED | 25-DEC-2022 02:02PM |
| | | PAPER ID | 94349736 |

# Association between coronary artery disease and incident cancer risk: a systematic review and meta-analysis of cohort studies

**Abstract**

**Objective:** Coronary artery disease (CAD) and cancer are the two leading causes of death worldwide. Evidence suggests the existence of shared mechanisms for these two diseases. We aimed to conduct a systematic review and meta-analysis to investigate association between CAD and incident cancer risk.

**Methods:** We searched Cochrane, PubMed, and Embase from inception until October 20, 2021, without language restrictions. Observational cohort studies were used to investigate the association between CAD and incident cancer risk. Using random-effects models, the odds ratio (OR) and 95% confidence interval (CI) were calculated. We utilized subgroup and sensitivity analyses to determine the potential sources of heterogeneity and explore the association between CAD and specific cancers. This study was conducted under a pre-established, registered protocol on PROSPERO (CRD42022302507).

**Results:** We initially examined 8,533 articles, and included 14 cohort studies in our review, 11 of which were eligible for meta-analysis. Patients with CAD had significantly higher odds of cancer risk than those without CAD (OR = 1.15, 95% CI = [1.08, 1.22], $I^2$ = 66%). Subgroup analysis revealed that the incident cancer risk was significantly higher in both sexes and patients with CAD with or without myocardial infarction. Sensitivity analysis revealed that the risk remained higher in patients with

CAD even after >1 year of follow-up (OR = 1.23, 95% CI = [1.08, 1.39], $I^2$ = 76%). Regarding the specific outcome, the incident risk for colorectal and lung cancers was significantly higher (OR = 1.06, 95% CI = [1.03, 1.10], $I^2$ = 10%, and OR = 1.36, 95% CI = [1.15, 1.60], $I^2$ = 90%, respectively) and that for breast cancer was lower (OR = 0.86, 95% CI = [0.77, 0.97], $I^2$ = 57%) in patients with CAD than in those without CAD.

**Conclusion:** CAD may be associated with incident cancer risk, particularly for lung and colorectal cancers, in men and women as well as patients with or without myocardial infarction. Early detection of new-onset cancer and detailed cancer surveillance programs should be implemented in patients with CAD to reduce cancer-related morbidity and mortality.

## Background

Cancers and coronary artery disease (CAD) are the two leading causes of death worldwide. They are closely associated with shared risk factors, which may indicate common biological characteristics, such as common pathways that result in smoking-related CAD and lung cancer.[1] Some studies have also suggested that cardiovascular diseases, such as myocardial infarction and cancer share similarities in terms of obesity, oxidative stress, and inflammation.[2, 3] People with mild CAD before cancer diagnosis may experience disease progression due to the cancer-induced proinflammatory and hypercoagulable states. Furthermore, CAD may cause a delay in the initiation of cancer treatment due to a decline in the patient's heart condition or increased risk of surgery.[1] Thus, early detection of neoplasm in patients with CAD through appropriate strategies is critical for reducing future morbidity.

Some studies have reported increased incidence of CAD and stroke after cancer diagnosis. Various radio- and chemotherapeutic agents may affect the development and progression of cardiovascular disease.[4-7] Further, several studies have indicated a high prevalence of occult cancer in patients with cardiovascular disease and reported that it is important to identify cancer risk factors as it may aid in developing new and effective preventive strategies.[8-10]

In contrast, several recent clinical and epidemiological studies have revealed a link between myocardial infarction and new-onset cancer;[11, 12] however, the findings

were inconsistent and contradictory.[13, 14] According to a systematic review, increased cancer risk after myocardial infarction was only significant in women and patients with certain cancers such as lung cancer. However, some of the review's analytic findings were based on only two or three studies and it only included patients with myocardial infarction, not all patients with CAD.[15] Recently, a large cohort study demonstrated that atherosclerotic cardiovascular disease itself increased cancer incidence after a median follow-up of 1,020 days.[16] Thus, the potential of CAD as a causal factor in cancer remains unknown. Furthermore, it has not yet been elucidated whether occult cancer occurs before the emergence of CAD. Therefore, this study aimed to conduct a comprehensive systematic review and meta-analysis to determine the association between CAD and incident cancer risk.

## Methods

*Data sources and study selection*

This systematic review followed the Preferred Reporting Items for Systematic Reviews and Meta-Analyses (PRISMA) guidelines (Table S1).[17] This protocol was registered into the PROSPERO International Prospective Register of Systematic Reviews (CRD42022302507).

The first author (Hsin-Hao Chen, HHC) and a medical librarian (Shu-Jung Liu, SJL) independently conducted an unrestricted search of electronic databases (Cochrane, PubMed, Embase [excluding Medline], and Taiwan Airiti Library) from inception until October 20, 2021. The following search terms were used: coronary artery disease, atherosclerosis, ischemic heart disease, myocardial infarction, neoplasms, cancer, and malignancy. The disagreements between the authors were resolved by a third reviewer (Tzu-Lin Yeh, TLY). We also examined potentially relevant studies in the references of relevant articles. Table S1 presents a complete description of the search strategies.

To identify eligible studies, we first removed duplicates. Two authors (Yi-Chi Lo, YCL and Wei-Sheng Pan, WSP) independently screened the titles and abstracts of each article, followed by a review of the full texts. If there was a disagreement, the third author (HHC) was consulted to reach consensus. Studies were included if they met the following criteria: (1) retrospective or prospective cohort studies; (2) studies

investigating the association between fatal or nonfatal CAD and cancer risk; (3) studies wherein cancer occurred after CAD diagnosis; and (4) studies reporting adjusted cancer relative risk (RR), odds ratio (OR), and hazard ratio (HR) with 95% confidence interval (CI). Further, the exclusion criteria were as follows: (1) animal studies; (2) cross-sectional and case–control studies wherein cancer may have occurred before or concurrently with CAD; (3) nonobservational article types; (4) studies that did not report the relevant data for extraction; or (5) literature reviews, republished data, case reports, dissertations, editorial, letter, or conference abstracts. We initiated the formal screening of search results while registering the protocol into PRSOPERO because we were afraid that the COVID-19 pandemic would affect the writing and review process at that time.

*Data extraction and quality assessment*

Two authors (YCL and WSP) independently extracted the following data from each included article: first author, publication year, publication country, study design, CAD type, number of enrolled participants, age, follow-up duration, adjusted factors, cancer type, and main results (Table 1). Any disagreements were resolved through discussion with the third author (HHC). If any information was missing from the study results, the authors of original studies were contacted via email. The Newcastle

Ottawa Scale (NOS)[18] was used by two authors (HHC and YCL) to independently assess the quality of the included studies. In cohort studies, the quality assessment tool (NOS) was used to rate each study in three domains—selection, comparability, and outcome—using a star system, with scores ranging from 0 to 9 stars.[19] The selection domain indicates representativeness of the exposed cohort, selection of the nonexposed cohort, and determination of exposure and outcome of interest that were absent at the beginning of the study. The comparability domain indicates whether exposed and nonexposed cohorts matched in the study design and/or whether confounders were adjusted for in the analysis. The outcome domain indicates whether the data were assessed accurately and whether the follow-up was adequate. If there was disagreement between two authors, the corresponding author (Tzu-Lin Yeh) made the final decision. A cohort study was considered to be of high quality if it received at least 6 stars.

*Statistical analysis and data synthesis*

We calculated pooled ORs with 95% CIs to estimate incident cancer risk in patients with CAD and compared it with that in patients without CAD. For our meta-analysis, we used statistical computing software R, version 4.1.2 (RStudio, Inc., Boston, MA, USA), primarily the Comprehensive R Archive Network package

"metagen."[20] Subsequently, we employed a random-effects model based on the DerSimonian and Laird's method with an assumption of nonidentical true effect sizes.[21] These results were presented as forest plots. Furthermore, heterogeneity among studies was quantified using Cochran's Q test and $I^2$ statistics, and a $p$-value of $<0.05$ in the Q test or $I^2$ value of $>50\%$ indicated the presence of heterogeneity.[22] Subgroup analysis was determine to assess the potential origins of heterogeneity. We did not perform a meta-regression analysis using patient characteristics, as some studies did not provide enough study-level variable information.[12, 23] Thus, this method would have been unsuitable, according to the methodological standards for meta-analysis and qualitative systematic reviews.[24] We investigated the association between CAD and different cancers, including lung, colorectal, breast, liver, and prostate cancers. To assess the robustness of the results, we performed a sensitivity analysis that included only studies with a follow-up time of >1 year. The risk of publication bias was assessed using funnel plots and Egger's test.[25]

**Results**

*Study characteristics and quality assessment*

Figure 1 presents the article selection flowchart. Initially, we obtained 8,533 articles from databases and by hand searching. Subsequently, we removed duplicates, reviewed titles and abstracts, and retrieved and evaluated 25 full-text articles for eligibility. After excluding articles with duplicate populations or those incompatible with the inclusion criteria, our systematic review included 14 cohort studies, 11 of which were eligible for meta-analysis (Fig.1).

Table 1 summarizes the general demographic characteristics of the included studies in the systematic review. Of the included studies, only two[16, 26] were conducted in Asia, whereas other studies were from USA or Europe. Four studies included patients with myocardial infarction identified via discharge diagnosis with Internal Classification of Disease (ICD) codes,[12-14, 27] whereas other studies included patients with CAD identified via hospital medical records, discharge diagnosis with ICD codes, or computed tomography scan with coronary artery calcium (CAC) score of >0. The duration of follow-up ranged from <1 year to a maximum of 33 years. Furthermore, we confirmed that the diagnosis of CAD was made before the occurrence of cancer in all included studies. Considering the cancer type, most studies investigated the incidence of all cancers, whereas other studies only assessed specific cancers, such as colorectal cancer,[23, 27] or cancers specific to men (prostate) or

women.[23, 28] Regarding the outcomes, a study only reported the incidence rate,[29] whereas other studies provided the overall or subgroup effect estimates of RR, OR, and HR with 95% CI.

In our study quality assessment, we observed that only one study did not report the items of selection and comparability domain and, as such, did not meet our criteria[11]. All other included studies received at least 6 of 9 stars on the NOS quality assessment scale, indicating high quality. Tables S3 presents the detailed results.

*Results of meta-analysis*

We pooled 11 studies for meta-analysis, which included >1,321,978 patients; however, one of these studies[12] did not specify the number of participants. Patients with CAD had significantly higher odds of cancer risk than those without CAD (OR = 1.15, 95% CI = [1.08, 1.22], $I^2$ = 66%; forest plot shown in Fig.2). Subgroup analyses were performed based on the heterogeneity in the country and CAD type of patients. Patients with CAD had significantly higher odds of cancer risk than those without CAD in non-Asian regions (OR = 1.15, 95% CI = [1.08, 1.23], $I^2$ = 67%; Fig.S1). Furthermore, Asian patients with CAD showed nonsignificantly higher odds of cancer risk than those without CAD (OR = 1.17, 95% CI = [0.89, 1.53], $I^2$ = 67%; Fig.S1). We also conducted a subgroup analysis by CAD subtype, which revealed that those with or without myocardial infarction had significantly higher odds of cancer risk

among patients with CAD than among those without CAD (OR = 1.11, 95% CI = [1.00, 1.23], $I^2$ = 89% and OR = 1.17, 95% CI = [1.08, 1.27], $I^2$ = 51%, respectively; Fig.S2).

*Subgroup analysis by sex*

We also performed pooled analyses in a random-effects model based on sex. This analysis was conducted when the studies indicated the odds of cancer risk by individual sex. After pooling seven studies, [11-14, 23, 28, 30] the overall risk of cancer incidence in men with CAD was higher than that in those without CAD (OR = 1.12, 95% CI = [1.03, 1.22], $I^2$ = 61%; Fig.3-1). Furthermore, after pooling six studies,[11-14, 23, 30] women with CAD showed a higher incident cancer risk than those without CAD (OR = 1.08, 95% CI = [1.00, 1.16], $I^2$ = 56%, Fig.3-2).

*Subgroup analysis by different outcome*

We determined whether CAD exerted different effects on different types of cancer. Patients with CAD had a significantly higher risk of colorectal and lung cancers than those without CAD (OR = 1.06, 95% CI = [1.03, 1.10], $I^2$ = 10%; Fig.4-1 and OR = 1.36, 95% CI = [1.15, 1.60], $I^2$ = 90%, respectively; Fig.4-2), as determined after pooling four [12, 26, 27, 30] and five[11-13, 26, 30] studies, respectively. However, according to the odds of breast cancer risk in five studies,[11-13, 26, 30] a lower risk was observed among patients with CAD than among those without CAD (OR = 0.86, 95%

CI = [0.77, 0.97], $I^2$ = 57%; Fig.4-3). Furthermore, compared with patients without CAD, a nonsignificantly increased risk of prostate and liver cancers was observed in those with CAD (OR = 1.04, 95% CI = [0.94, 1.16], $I^2$ = 72%; Fig.S3-1 and OR = 1.03, 95% CI = [0.88, 1.21], $I^2$ = 59%, respectively; Fig.S3-2), as determined after pooling seven[11-13, 23, 26, 28, 30] and three[11, 12, 26] studies, respectively.

*Sensitivity analysis and publication bias*

We analyzed six studies in which all patients had a follow-up time of >1 year.[14, 23, 26, 27, 31, 32] The incident cancer risk was still higher in patients with CAD than in those without CAD (OR = 1.23, 95% CI = [1.08, 1.39], $I^2$ = 76%; Fig.S4). Funnel plots revealed asymmetry for publication bias, as shown in Fig.S5. In addition, Egger's test revealed a significant publication bias ($p = 0.06$).

**Discussion**

Our meta-analysis revealed that patients with CAD had significantly higher odds of cancer risk than those without CAD among cohort studies. Subgroup analysis indicated that cancer risk was significantly higher in both men and women, those with and without myocardial infarction, and non-Asian patients. Moreover, for specific cancer types, patients with CAD had a higher risk of colorectal and lung cancers, nonsignificantly higher risk of prostate and liver cancers, and lower risk of breast cancer.

A previous systematic review of myocardial infarction based on only three studies revealed that the incident cancer risk in the test group was nonsignificantly higher (OR = 1.08, 95% CI = [0.97, 1.19]) than that in the control group. However, subgroup analysis revealed that the overall cancer risk was higher in women and during the first 6 months following myocardial infarction diagnosis.[15] Further, our meta-analysis of eleven studies revealed a significantly higher incident cancer risk in patients with CAD with or without myocardial infarction. One of the differences in the outcomes of patients with myocardial infarction is the number of cohort participants included in the meta-analysis. As the 1998 study by Dreyer in Denmark[11] comprised only a small proportion (96891 people) of the 2013 study by Erichsen (297523 people),[27] we included a large cohort instead of a small cohort. Further, our

meta-analysis evaluated patients without myocardial infarction via CAC, percutaneous coronary intervention (PCI), or hospital discharge records to comprehensively assess cancer risk in patients with CAD.

CAD and incident cancer risk are mainly associated because of the presence of shared risk factors. As summarized in the study by Hasin et al., cancer may be caused by treatment modalities or biological changes related to cardiovascular diseases.[33] Other reviews have also indicated that inflammatory cytokines, such as interleukin(IL)-1, IL-6, IL-10, tumor necrosis factor-α, macrophage migration inhibitory factor, and transforming growth factor-β, are involved in tumor initiation and progression.[34, 35] In addition to inflammation during the development of atherosclerosis and cancer, a recent review revealed that age-related mutations, obesity, smoking, and diabetes are overlapping risk factors between cancer and CAD.[35] Additionally, some observational studies have reported that noncardiac causes, such as malignancies, are responsible for most later deaths in patients with myocardial infarction treated with PCI.[36, 37]

Conversely, some studies have suggested that the increased cancer risk immediately after myocardial infarction can be attributed to other confounding factors, such as surveillance bias, rather than myocardial infarction itself. Patients with myocardial infarction had frequent clinical appointments and underwent more

diagnostic examinations, especially in the first few months after the event, which may increase the likelihood of early cancer detection.[13, 15] This situation is not only observed in patients with myocardial infarction but also in those without. Other studies have shown that occult cancers could have occurred before the cardiovascular event if cancer incidence is observed immediately after the start of myocardial infarction follow-up.[38] In some patients, an underlying malignancy can cause an ischemic stroke. The effects of the coagulation cascade, tumor mucin secretion, infections, and nonbacterial endocarditis may contribute to the mechanisms.[39] Thus, occult cancer may also contribute to the development of CAD. However, our sensitivity analysis revealed that patients with CAD continue to have an increased incident cancer risk after >1 year of follow-up, which differs from the meta-analysis based on only two studies reporting that cancer risk is only significant in the first 6 months. Another study revealed that although the cancer risk is the highest in the first year following myocardial infarction, cancer develops over time.[13] According to a recent large-scale cohort study, atherosclerotic cardiovascular disease increases the incident cancer risk after a median follow-up of 1,020 days.[16] Moreover, the risk is increased when patients with CAD concomitantly have aortic and peripheral artery disease with a median follow-up of 3 years.[40] Therefore, CAD may affect long-term cancer incidence.

Our study revealed that CAD events increased the risk of lung and colorectal cancers but decreased the risk of breast cancer. We determined that “smoking,” a well-known cause of lung and colorectal cancers, was a common risk factor. This may account for some of our findings that indicate that the risk of both cancers was significantly increased after CAD.[41] Another reason for an increase in lung cancer incidence may be that cardiac scanning includes the lungs; thus, lung cancers account for most detected cancers.[30] Diabetes is a classic risk factor for CAD and is also related to elevated risk of cancer, especially colorectal cancer.[35] A study showed that patients with diabetes had a 20%–38% higher cancer risk than those without diabetes.[42] Moreover, modifiable environmental risk factors, such as obesity, lack of physical activity, and westernized diet, may predispose individuals to CAD and colorectal cancer.[43] According to two large prospective cohort studies, a high intake of animal fat or processed red meat and low intake of fiber could increase the risk of CAD and colon cancer.[44, 45] One possible explanation for the lower risk of breast cancer in our study is life-long aspirin treatment, as recommended by CAD guidelines,[46] which may also affect carcinogenesis. Large-scale cohort studies have consistently demonstrated the protective effects of low-dose aspirin for treating breast cancers.[46, 47] However, there is limited evidence to support the association between CAD and breast cancer and we cannot exclude the possible selection bias; therefore,

more research is warranted in this regard.

This is the first study to conduct a comprehensive review and meta-analysis of the association between CAD and incident cancer risk with regard to patients with or without myocardial infarction as well as different cancer types. However, there are some limitations that must be addressed. First, our meta-analysis had significant publication bias, indicating that some nonsignificant studies are not published. This would weaken the positive association between CAD and incident cancer risk observed in our study. However, current evidence was the best available, and all studies, including several population-based cohort studies, were of moderate-to-high quality. Second, not all included studies could distinguish the length of follow-up and different cancer types. Our findings showed that the cancer risk remains elevated even at 1 year of follow-up after a CAD event, which contradicts the findings of the previous two studies.[13, 26] According to our subgroup analysis, CAD may have different effects on different cancer types. Additional studies with subgroup analysis of follow-up time and different types of cancer are thus warranted to investigate the association between CAD and incident cancer risk. Third, most studies did not provide data regarding heart failure or left ventricular ejection fraction. Recently, Meijers et al. indicated that heart failure stimulates tumor growth via cardiac-excreted circulating factors.[48] Furthermore, heart failure is associated with cancer incidence [33]

and could become a confounding factor in future research.

## Conclusions

Based on our analysis of newly published data, we observed an increased risk of incident cancer after a CAD event. This was observed in men and women as well as patients with cancers, particularly lung and colorectal cancers, with or without myocardial infarction. Although this trend may be attributable to several common risk factors and underlying pathophysiologic mechanisms such as inflammation, patients with a history of CAD are still more likely to develop cancer. As CAD and cancer are the two leading causes of death, treatment of any one disease may affect the occurrence of the other. Therefore, more research is warranted regarding the causes of malignancy. Further, detailed cancer surveillance and possible interventions in the CAD population should be implemented to reduce cancer-related morbidity and mortality.

## Acknowledgments

We would like to thank the MacKay Memorial Hospital librarian, Pei-jin Li, for examining the references. We would like to thank Romy E (www.enago.tw) for editing and proofreading the manuscript. The research did not receive any specific grants from funding agencies in the public, commercial or not-for-profit sectors.

**Competing interests statement**

All authors declare that there is no conflict of interest regarding the publication of this study.

**Data availability**

The datasets during and/or analyzed during the current study is available as attached file.

Reference

1. Das D, Asher A, Ghosh AK. Cancer and Coronary Artery Disease: Common Associations, Diagnosis and Management Challenges. *Curr Treat Options Oncol* 2019;20:46.

2. Koene RJ, Prizment AE, Blaes A, Konety SH. Shared Risk Factors in Cardiovascular Disease and Cancer. *Circulation* 2016;133:1104-14.

3. Pischon T, Nimptsch K. Obesity and Risk of Cancer: An Introductory Overview. *Recent Results Cancer Res* 2016;208:1-15.

4. Arthurs E, Hanna TP, Zaza K, Peng Y, Hall SF. Stroke After Radiation Therapy for Head and Neck Cancer: What Is the Risk? *Int J Radiat Oncol Biol Phys* 2016;96:589-96.

5. Navi BB, Reiner AS, Kamel H, Iadecola C, Elkind MS, Panageas KS, et al. Association between incident cancer and subsequent stroke. *Ann Neurol* 2015;77:291-300.

6. Zöller B, Ji J, Sundquist J, Sundquist K. Risk of coronary heart disease in patients with cancer: a nationwide follow-up study from Sweden. *Eur J Cancer* 2012;48:121-8.

7. Zhang F, Wang K, Du P, Yang W, He Y, Li T, et al. Risk of Stroke in Cancer Survivors: A Meta-analysis of Population-Based Cohort Studies. *Neurology*

2021;96:e513-e26.

8. Corraini P, Szépligeti SK, Henderson VW, Ording AG, Horváth-Puhó E, Sørensen HT. Comorbidity and the increased mortality after hospitalization for stroke: a population-based cohort study. 2018;16:242-52.

9. Tybjerg AJ, Skyhøj Olsen T, Andersen KK. Prevalence and risk of occult cancer in stroke. *Acta neurologica Scandinavica* 2020;141:204-11.

10. Wang FM, Reiter-Brennan C, Dardari Z, Marshall CH, Nasir K, Miedema MD, et al. Association between coronary artery calcium and cardiovascular disease as a supporting cause in cancer: The CAC consortium. *Am J Prev Cardiol* 2020;4:100119.

11. Dreyer L, Olsen JH. Cancer risk of patients discharged with acute myocardial infarct. *Epidemiology* 1998;9:178-83.

12. Pehrsson SK, Linnersjö A, Hammar N. Cancer risk of patients with ischaemic syndromes. *J Intern Med* 2005;258:124-32.

13. Malmborg M, Christiansen CB, Schmiegelow MD, Torp-Pedersen C, Gislason G, Schou M. Incidence of new onset cancer in patients with a myocardial infarction - a nationwide cohort study. *BMC Cardiovasc Disord* 2018;18:198.

14. Rinde LB, Smabrekke B, Hald EM, Brodin EE, Njolstad I, Mathiesen EB, et al. Myocardial infarction and future risk of cancer in the general population-the Tromso Study. *Eur J Epidemiol* 2017;32:193-201.

15. Li N, Huang Z, Zhang Y, Sun H, Wang J, Zhao J. Increased cancer risk after myocardial infarction: fact or fiction? A systemic review and meta-analysis. *Cancer Manag Res* 2019;11:1959-68.

16. Suzuki M, Tomoike H, Sumiyoshi T, Nagatomo Y, Hosoda T, Nagayama M, et al. Incidence of cancers in patients with atherosclerotic cardiovascular diseases. *Int J Cardiol Heart Vasc* 2017;17:11-6.

17. Page MJ, McKenzie JE, Bossuyt PM, Boutron I, Hoffmann TC, Mulrow CD, et al. The PRISMA 2020 statement: an updated guideline for reporting systematic reviews. *Bmj* 2021;372:n71.

18. GA Wells, B Shea, D O'Connell, J Peterson, V Welch, M Losos, P Tugwell. The Newcastle-Ottawa Scale (NOS) for assessing the quality of nonrandomised studies in meta-analyses. Ottawa Hospital Research Institute. Available at: https://www.ohri.ca/programs/clinical_epidemiology/oxford.asp. Accessed December 20, 2022.

19. Zeng X, Zhang Y, Kwong JS, Zhang C, Li S, Sun F, et al. The methodological quality assessment tools for preclinical and clinical studies, systematic review and meta-analysis, and clinical practice guideline: a systematic review. *J Evid Based Med* 2015;8:2-10.

20. Software R. Available at: https://www.rstudio.com/products/rstudio/download/.

Accessed September 1, 2022.

21. DerSimonian R, Laird N. Meta-analysis in clinical trials. *Control Clin Trials* 1986;7:177-88.

22. Higgins JP, Thompson SG. Quantifying heterogeneity in a meta-analysis. *Stat Med* 2002;21:1539-58.

23. Dzaye O, Berning P, Dardari ZA, Mortensen MB, Marshall CH, Nasir K, et al. Coronary artery calcium is associated with increased risk for lung and colorectal cancer in men and women: the Multi-Ethnic Study of Atherosclerosis (MESA). *European heart journal Cardiovascular Imaging* 2021.

24. Rao G, Lopez-Jimenez F, Boyd J, D'Amico F, Durant NH, Hlatky MA, et al. Methodological Standards for Meta-Analyses and Qualitative Systematic Reviews of Cardiac Prevention and Treatment Studies: A Scientific Statement From the American Heart Association. *Circulation* 2017;136:e172-e94.

25. Egger M, Davey Smith G, Schneider M, Minder C. Bias in meta-analysis detected by a simple, graphical test. *Bmj* 1997;315:629-34.

26. Kwak S, Choi YJ, Kwon S, Lee SY, Yang S, Moon I, et al. De novo malignancy risk in patients undergoing the first percutaneous coronary intervention: A nationwide population-based cohort study. *Int J Cardiol* 2020;313:25-31.

27. Erichsen R, Sværke C, Sørensen HT, Sandler RS, Baron JA. Risk of colorectal

cancer in patients with acute myocardial infarction and stroke: a nationwide cohort study. *Cancer Epidemiol Biomarkers Prev* 2013;22:1994-9.

28. Thomas JA, Gerber L, Bañez LL, Moreira DM, Rittmaster RS, Andriole GL, et al. Prostate cancer risk in men with baseline history of coronary artery disease: results from the REDUCE Study. *Cancer epidemiology, biomarkers & prevention* 2012;21:576-81.

29. Berton G, Cordiano R, Cavuto F, Bagato F, Segafredo B, Pasquinucci M. Neoplastic disease after acute coronary syndrome: incidence, duration, and features: the ABC-4* Study on Heart Disease. *J Cardiovasc Med (Hagerstown)* 2018;19:546-53.

30. Vinter N, Christesen AMS, Mortensen LS, Lindholt JS, Johnsen SP, Tjønneland A, et al. Relation of Coronary Artery Calcium Score and Risk of Cancer (from a Danish Population-Based Follow-up Study in Patients Who Underwent Cardiac Computed Tomography). *Am J Cardiol* 2017;120:542-9.

31. Handy CE, Desai CS, Dardari ZA, Al-Mallah MH, Miedema MD, Ouyang P, et al. The Association of Coronary Artery Calcium With Noncardiovascular Disease: The Multi-Ethnic Study of Atherosclerosis. *JACC Cardiovasc Imaging* 2016;9:568-76.

32. Peng AW, Mirbolouk M, Orimoloye OA, Osei AD, Dardari Z, Dzaye O, et al.

Long-Term All-Cause and Cause-Specific Mortality in Asymptomatic Patients With CAC ≥1,000: Results From the CAC Consortium. *JACC Cardiovasc Imaging* 2020;13:83-93.

33. Hasin T, Gerber Y, Weston SA, Jiang R, Killian JM, Manemann SM, et al. Heart Failure After Myocardial Infarction Is Associated With Increased Risk of Cancer. *J Am Coll Cardiol* 2016;68:265-71.

34. Amin MN, Siddiqui SA, Ibrahim M, Hakim ML, Ahammed MS, Kabir A, et al. Inflammatory cytokines in the pathogenesis of cardiovascular disease and cancer. *SAGE Open Medicine* 2020;8.

35. Leiva O, AbdelHameid D, Connors JM, Cannon CP, Bhatt DL. Common Pathophysiology in Cancer, Atrial Fibrillation, Atherosclerosis, and Thrombosis: JACC: CardioOncology State-of-the-Art Review. *JACC CardioOncol* 2021;3:619-34.

36. Pedersen F, Butrymovich V, Kelbæk H, Wachtell K, Helqvist S, Kastrup J, et al. Short- and long-term cause of death in patients treated with primary PCI for STEMI. *J Am Coll Cardiol* 2014;64:2101-8.

37. Spoon DB, Psaltis PJ, Singh M, Holmes DR, Jr., Gersh BJ, Rihal CS, et al. Trends in cause of death after percutaneous coronary intervention. *Circulation* 2014;129:1286-94.

38. Hasin T, Iakobishvili Z, Weisz G. Associated Risk of Malignancy in Patients

with Cardiovascular Disease: Evidence and Possible Mechanism. *Am J Med* 2017;130:780-5.

39. Selvik HA, Thomassen L, Bjerkreim AT, Næss H. Cancer-Associated Stroke: The Bergen NORSTROKE Study. *Cerebrovasc Dis Extra* 2015;5:107-13.

40. Suzuki M, Tomoike H, Dai Z, Hosoda T, Sumiyoshi T, Hosoda S, et al. Polyvascular Disease and the Incidence of Cancer in Patients with Coronary Artery Disease. *Jma j* 2022;5:498-509.

41. Dekker E, Tanis PJ, Vleugels JLA, Kasi PM, Wallace MB. Colorectal cancer. *Lancet* 2019;394:1467-80.

42. Yuhara H, Steinmaus C, Cohen SE, Corley DA, Tei Y, Buffler PA. Is diabetes mellitus an independent risk factor for colon cancer and rectal cancer? *Am J Gastroenterol* 2011;106:1911-21.

43. Keum N, Giovannucci E. Global burden of colorectal cancer: emerging trends, risk factors and prevention strategies. *Nat Rev Gastroenterol Hepatol* 2019;16:713-32.

44. Al-Shaar L, Satija A, Wang DD, Rimm EB, Smith-Warner SA, Stampfer MJ, et al. Red meat intake and risk of coronary heart disease among US men: prospective cohort study. *Bmj* 2020;371:m4141.

45. Willett WC, Stampfer MJ, Colditz GA, Rosner BA, Speizer FE. Relation of meat, fat, and fiber intake to the risk of colon cancer in a prospective study among

women. *N Engl J Med* 1990;323:1664-72.

46. Qiao Y, Yang T, Gan Y, Li W, Wang C, Gong Y, et al. Associations between aspirin use and the risk of cancers: a meta-analysis of observational studies. *BMC Cancer* 2018;18:288.

47. Yang YS, Kornelius E, Chiou JY, Lai YR, Lo SC, Peng CH, et al. Low-Dose Aspirin Reduces Breast Cancer Risk in Women with Diabetes: A Nationwide Retrospective Cohort Study in Taiwan. *Journal of women's health (2002)* 2017;26:1278-84.

48. Meijers WC, Maglione M, Bakker SJL, Oberhuber R, Kieneker LM, de Jong S, et al. Heart Failure Stimulates Tumor Growth by Circulating Factors. *Circulation* 2018;138:678-91.

49. Mirbolouk M, Kianoush S, Dardari Z, Miedema MD, Shaw LJ, Rumberger JA, et al. The association of coronary artery calcium score and mortality risk among smokers: The coronary artery calcium consortium. *Atherosclerosis* 2020;294:33-40.

**Figure title and legends**

**Figure 1**. Flowchart for selection of articles

**Figure 2**. Forest plot of comparing incident cancer risk in patients with CAD with that in patients without CAD; CI, confidence interval; OR, odds ratio; se, standard error; TE, treatment effect.

**Figure 3**. Forest plot of comparing incident cancer risk in patients with CAD with that in patients without CAD by individual sex. CAD, coronary artery disease; CI, confidence interval; OR, odds ratio; se, standard error; TE, treatment effect.

**Figure 4.** Forest plot of comparing incident cancer risk in patients with CAD with that in patients without CAD by individual cancer type. CAD, coronary artery disease; CI, confidence interval; OR, odds ratio; se, standard error; TE, treatment effect.

# Association between coronary artery disease and incident cancer risk: a systematic review and meta-analysis of cohort studies

ORIGINALITY REPORT

3%
SIMILARITY INDEX

PRIMARY SOURCES

| | | |
|---|---|---|
| 1 | hdl.handle.net<br>Internet | 26 words — 1% |
| 2 | Yifan Xu, Zhuang Wu, Hang Xu. "Cancer-related cryptogenic stroke involving the bilateral anterior and the posterior circulations: Diagnostic value of clinical and imaging characteristics", Frontiers in Neurology, 2022<br>Crossref | 25 words — 1% |
| 3 | link.springer.com<br>Internet | 25 words — 1% |
| 4 | Valentina Milazzo, Nicola Cosentino, Jeness Campodonico, Claudia Lucci, Daniela Cardinale, Carlo M. Cipolla, Giancarlo Marenzi. "Characteristics, Management, and Outcomes of Acute Coronary Syndrome Patients with Cancer", Journal of Clinical Medicine, 2020<br>Crossref | 24 words — 1% |
| 5 | pubmed.ncbi.nlm.nih.gov<br>Internet | 21 words — 1% |

EXCLUDE QUOTES ON

EXCLUDE SOURCES < 1%

EXCLUDE BIBLIOGRAPHY ON EXCLUDE MATCHES < 5 WORDS
